# Supplementary material for: Getting to the Emergency Department in time: Interviews with patients and their caregivers on the challenges to emergency care utilization in rural Uganda
Source: PLoS One. 2022 Aug 4;17(8):e0272334. doi: 10.1371/journal.pone.0272334 (PMC9352071; doi:10.1371/journal.pone.0272334)
Supplement: Attachment — Submitted filename: Response to Reviewers.docx [file pone.0272334.s004.docx]

Catherine A Staton, M.D., MSc

*Academic Editor*

*PLOS ONE*

August 20, 2021

Dear Dr Staton,

Thank you for your in-depth review of our work. We are thrilled that the reviewers find this work to be as important as we do. The insights and reviewer comments helped to clarify and strengthen the paper.

Overall, reviewer comments helped us to clarity the scope of our work both internally and for the reader. The aim of this study was increasing understanding of the challenges and delays to EC access in the community. While we are aware that there is significant work to be done to facilitate timely, quality emergency care at the health facility level this was not the focus of our aims or methods. Ultimately, this study is an initial formative work seeking to understand the emergency care seeking behavior of community members utilizing the established ED. As such the focus was on understanding challenges to emergency care access and not on specific interventions to address these challenges which would be acceptable to and effective for the community. While we make several general recommendations for areas of focus for ongoing research on interventions, this is not the focus of our paper. Rather, as this one of the first studies to describe challenges to seeking care at an established emergency department we contextualize our results within available literature on care seeking in acute illness and injury, drawn largely from the pediatrics and obstetrics literature. We believe that this increases the value of our results in adding to understanding of emergency care seeking behavior and in providing a basis for ongoing research. We have clarified our aims within the discussion section and edited our title to help the reader better understand the scope of the work. We integrated the three delays model into the discussion section and added additional discussion around the support our results lend to the model’s applicability to emergency care access.

We utilized the COREQ guideline in conducting our study and drafting our paper to ensure compressive reporting. This has been added to the methods section.

Our IRB does not allow for data sharing, so we will be unable to publish the interview transcripts. However, as suggested we have added a supplement including our code book with further representative quotes to ensure readers have as broad an access as ethically possible.

We have addressed all reviewer comments inline below.

We look forward to your review of our revisions and final decision regarding publication.

Thank you,

Ashley Pickering, MD, MPH

Corresponding Author

University of Maryland Medical Center

Department of Emergency Medicine

110 S. Paca St, Suite 600

Baltimore, MD 21201

Department Phone: 667-214-2181

Reviewer comments addressed in-line (line numbers refer to markup):

Reviewer #1: 1.Is the manuscript technically sound, and do the data support the conclusions?
Overall a well written manuscript on an important topic. I would suggest considering adding additional quotes to support your findings.

*Several additional quotes were added to the results section. We also added supplemental information containing our codebook with many additional quotes by subtheme.*

2. Has the statistical analysis been performed appropriately and rigorously?
Methodologically sound. Mention in your methods what reporting guideline was used for this manuscript.

*Added line 286/287 - The Consolidated Criteria for Reporting Qualitative Research (COREQ) was utilized to ensure comprehensive reporting.*

3. Have the authors made all data underlying the findings in their manuscript fully available?
You state that the data is available in the supporting files, however this is only the codebook and not the transcripts. If the IRB approves releasing the full transcripts, that would qualify as all data is fully available without restriction.
If not, explain the reasoning for restricted/no access, and consider adding quotes to the full codebook in the supporting information.

*As above - Our IRB does not allow for data sharing, so we will be unable to publish the interview transcripts. However, as suggested we have added a supplement including our code book with further representative quotes.*

Overall comments:
Interesting and important study, with robust qualitative methods, and integrating ongoing local efforts to increase access to EC.
Discussion could benefit from some minor restructuring. Lots of re-iteration of the results. The three delay framework gets introduced, but throughout the discussion it is not clear how it was applied. Consider adding the headings from the identified delays you are discussing, and how those could be addressed (the 4 main points for action you mention in the Conclusion)

*These changes were made in the discussion section. The Three Delays model was applied as a framework for structuring discussion and contextualization of key results (as is common in most discussion sections) within a review of the limited available literature and ideas of how these could be addressed were moved from the conclusions to the discussion.*

It is not clear to me from this paper why community based interventions should be the next focus, instead of provider centered interventions and Health systems strategies.

*As above - The aim of this study was increasing understanding of the challenges and delays to EC access in the community. While we are aware that there is significant work to be done to facilitate timely, quality emergency care at the health facility level this was not the focus of our aims or methods.*

The Corresponding and first author is affiliated with GEC, the setting the interviews took place and patients were treated, and was present during the interviews. How was a potential power dynamic between the interviewer team and patients managed, so that participants did not feel pressured to participate?

*Added line 242-244 - AP was not involved in providing clinical care. NC approached patients to gauge interest in study participation, limiting pressure to participate.*

Feedback by line:
Line 36: How was data saturation determined?

*This statement as deleted as the abstract is not the appropriate word count for an in-depth discussion of data saturation.*

*Question is addressed 282-283 -* *Data saturation was determined by additional interviews failing to demonstrate additional themes of delay.*

Line 40-42: Does this include patient and caregivers? The remainder of the participant characteristics is centered around the patients. It would be helpful to separate gender and occupation for patient and caregivers

*Addressed 65-67: The 50 ED patients for whom care was sought (mean age 33) with approximately even distribution of gender, as well as occupation (none, subsistence farmers and business owner).*

Line 40: How have you identified that interventions in the community are necessary instead of possible HS based interventions/ provider interventions etc.

*Addressed line 90 – 92*: Interventions are warranted to address each of the four major reasons for treatment delay. The next stage of formative research will generate intervention strategies and assess the opportunities and challenges to implementation with community and health system stakeholders

Line 66: remove brackets,

*Removed*

Line 75-83: Good summary of the need for action/ research

*Thank you*

103-107: references available?

*Reference added*

96-123: What information is essential for the reader to understand your research? Consider shortening this section

*Removed non-essential data*

Line 139: Why >12 hours after presenting to the ED?

*≥12 hours was set as a benchmark based on advice from the Ugandan clinicians working in the ED*

Line 141: How was determined who has significant experience with challenges in EC access and utilization?

*Addressed lines 205/206 - based on conversations with ED staff*

Line 154: Was assent obtained from the patients, if the caregivers were interviewed on their behalf, since the interview questions were directly related to the patient experience?

*Addressed lines 220-221: In the case of caregiver interviews, assent was obtained from patients old enough, or alert enough to provide it. In most instances this applied to the young children, the elderly and those with severe illness.*

164: add the reference to the three delays model

*Reference added*

166/67: some additional detail on the translation process and validation. How was consensus reached?

*Addressed lines 250-252 - If there was a discrepancy in translation/interpretation the audio recording was reviewed by NC and KA together and discussed until consensus was reached.*

179: Why are patients utilizing the ED at the time of interview not appropriate for member checking? Please clarify

*Addressed lines 254-255: Due to the nature of the sample — patients utilizing the ED — typically participants were not available on site when transcription and translation were complete. The IRB limited collection of contact information to ensure privacy, therefore transcripts were not returned to interviewees for review.*

186: growing field of research now. Consider clarifying, that in 2017, little was known about delays in accessing care

*Added this clarification on line 261-262*

195: refer to the supporting information or where the codebook can be found

*Codebook with additional quotes included as Appendix S2*

203: Are some caregivers of patients also included in the study? Highlight the most important characteristics in text instead of just referring to the table.

*Addressed lines 290-298- The 50 ED patients for whom care was sought (13 of whom were interviewed directly) had a mean age of 33 years. Patients had an approximately even distribution of gender, as well as occupation (none, subsistence farmers and small business owner). Demographics and CCs for the ED patients are illustrated in Table 1. A full list of CCs is included as Appendix S3. Fifty interviews were completed, 37 with caregivers of ED patients and 13 with the ED patients themselves. Interviewees had a mean age of 38 years, and the majority were women, worked as subsistence farmers, and had received primary education. The demographics of interview participants are presented in Table 2.*

206: Table 1: Not immediately clear what the participant sample looks like. Consider splitting the table up in patient and caregiver characteristics

*Clarified relationship of interview participant to patient. Switched the ordered or Tables 1 and 2 to help clarify the demographics for all patients, prior to discussing the overlap between patients and caregivers.*

207: Table 2: What are other medical complaints? (Given that it is 26% of all medical cases.

*Added all list of all chief complaints in Appendix S3*

234: remove “a”

*Removed*

260: is that a quote? “Failed” How were failed treatments perceived by the participants?

*Addressed line 274 - treatment is thought to have been ineffective*

264: if 8$ is a large sum, provide some context, eg average net income

*As address in the introduction, subsection The Rukungiri District, 78% of the population are subsistence farmers. We have not been able to identify any source of average net income for the district, the western region, or even rural areas of Uganda. Overall average income for Ugandan is significantly higher than in the Rukungiri District due to the impact of urban areas with increased individuals with formal employment.*

286: was this data provided by the participants during the interview? Consider clarifying that this is based on their recall and no additional information sources.

*Clarification added lines 444-448 - based on the distance from the center of the identified parish to the hospital determined through GIS measurement for a prior study (23)*

295: What is meant with opportunity costs?

*Clarification added lines 457-458–* *The opportunity costs of health care seeking on work and ability to perform essential household activities impacted the decision to seek care*

344: Was the interview guide developed based on the three delay model or applied afterwards?

*Addressed line 510-511 - The interview guide and a framework for the themes we identified were informed by the Three Delays Model*

347: Figure 1: make sure to demonstrate which your own themes of delay are based on the data.

*Figure updated to clarify*

Reviewer #2: The authors present an important manuscript qualitatively evaluating reasons for delays in seeking emergency care in rural Uganda as elicited from patients and caregivers. A sound framework and underlying theoretical model were used.
- 50 surveys for a qualitative project is a large number, and data saturation was already reached at 25. Were there any additional themes identified in the last 25?

*Addressed lines 270-282:* These themes and subthemes constituted a codebook, included as Appendix S2, facilitating coding of an additional 25 interviews completed to capture a broader sample of demographic groups and chief complaints across both dry and rainy seasons, given differences in EC accessibility and subsistence agriculture activities. Data saturation was determined when interviews failed to demonstrate additional themes related to treatment delay.

- Table 2 separates CC by medical and injury, yet in table 3 and elsewhere, CC is separated by medical illness, injury, and chronic disease. This should be consistent across tables.

*We separated CC by medical and injury. History of chronic disease was found to significantly increase delay for acute illness or injury, regardless of if it was related to the chronic disease, so this data as pulled on in table 3 to illustrate this finding.*

- Further clarification of chief complaint is needed. The authors indicate that only acute presentation of chronic conditions were included in the sample, however, in Table 3 there are chief complaints that have a duration of 3 years noted. This does not make sense for an acute presentation of an illness or a medical illness.

*Addressed lines 314-320 - The most notable trend related to treatment delay at the ED was history of chronic disease. ED patients with acute presentations and history of underlying chronic conditions experienced substantially longer delays in reaching the ED than otherwise healthy patients. Among chronically ill patients, recognition of acute symptoms needing EC ranged from 12 hours – 14 days prior to arriving at the ED, with an average of 6 days. For all patients included in the study the mean duration from recognition of symptoms necessitating EC to presentation at the ED as 1 day. Patients with chronic disease and their caregivers reported challenges to decision-making including perceptions that:*

- Given the distinct differences in trauma and medical emergency complaints, it would be interesting to note if there were any differences in subthemes between these 2 groups. For example, "cultural illnesses" requiring treatment by a traditional healer, seem to be more for primarily medical emergency conditions.

*Any differences identified have been highlighted in the paper*

- Limitations section notes subjectivity of the data on the quality of local clinics. This is not a limitation and should be removed. Given that this is qualitative research, these are the noted perceptions of the interviewees and important as such in terms of understanding behaviors behind using or not using these services.

*This was removed from the limitations and is addressed in the discussion section.*

Other errors:
Line 164: need to cite The Three Delays model

*Citation added.*

Line 265: missing a period after ED

*Addressed.*

Line 351: missing the word "needing" before emergency treatment?

*Addressed.*

Reviewer #3: The goal of the study was to assess/explore the barriers for accessing and utilizing emergency care in rural Uganda. Which I think the study achieved. It was done by using semistructured interviews. 50 patients and caregivers were interviewed. Good description of method with construction of the interview questions, how the authors tried to avoid bias, ethical approvoals etc. Four major themes of delay appeared: 1) "Limited knowledge of emergency signs and of initial actions to take"; 2) "Use of local facilities for stabilization and advice despite perception of in adequate services"; 3) "Lack of resources to cover the direct, indirect and opportunity costs of EC"; 4) "In adequate transportation options, especially at night and inclement weather". In the discussion the theory of the Three Delays model (where focus was on the two first ones) was discussed and applied to the results and where a correlation was found which I find was good. Four issues (1) teaching programs, 2) EC loans, 3) emergency transport and communicating systems, 4) referral system) are pointed out in the conclusion that needs immediate attention which are all good and in line with the findings but I would have liked them to have been motivated a little bit more and maybe already pointed out in the discussion section.

*The four broad intervention strategies identified by the reviewer were moved to the discussion section. We provide limited discussion of interventions as this is largely outside the scope of this work and requires further research is addressed in the letter above.*
