# Supplementary material for: Getting to the Emergency Department in time: Interviews with patients and their caregivers on the challenges to emergency care utilization in rural Uganda
Source: PLoS One. 2022 Aug 4;17(8):e0272334. doi: 10.1371/journal.pone.0272334 (PMC9352071; doi:10.1371/journal.pone.0272334)
Supplement: Attachment — Submitted filename: Response to reveiwers 5_22.docx [file pone.0272334.s005.docx]

Reviewer Comments to the Author

Please use the space provided to explain your answers to the questions above. You may also include additional comments for the author, including concerns about dual publication, research ethics, or publication ethics. (Please upload your review as an attachment if it exceeds 20,000 characters)

Reviewer #1: Thank you for addressing our comments to the previous version of the manuscript. I enjoyed reading the article and think you have made the aims of this project and placing it into the larger research focus of your team much clearer. The integration of the three-delay framework and discussion especially are very well written.
I think the abstract is currently not reflecting the changes you made to the manuscript appropriately. Additionally, I suggest some restructuring and clarifications in the methods section, to further increase the rigor of the study. Please see more specific suggestions below.

Line5-7: In the response letter to the editor, you very clearly state the aims of this study. I suggest using that in the abstract. “Implementation of effective EC requires assessment of socioeconomic, cultural, and structural factors leading to treatment delay.” Might lead the reader to believe you will be focusing on the implementation of strategies, instead of first understanding the challenges.

*Line 6-7 changed to: “*This study seeks to understand the emergency care seeking behavior of community members utilizing the established ED.”

Line 13-14: not clear how translating and transcribing the interviews allow for a grounded theory approach and thematic analysis. I’d suggest simply stating what analysis approach you chose. If there is space, I suggest stating why you chose 50 participants as a sample size or if it was driven by saturation.

Addressed on lines 11-14: “Semistructured interviews addressing actions taken before seeking EC and delays to presentation once the need for EC was recognized were conducted until theoretical saturation and a diverse sample were obtained. An interdisciplinary and multicultural research team conducted thematic analysis based on descriptive phenomenology.”

18: I know space is very limited and you address it later in text, but some readers might wonder why there are almost three times as many caregivers interviewed as patients? Were they used as a proxy because the patients were not available? Could be as simple as stating “patients or caregivers of patients being unavailable were interviewed on their behalf.”
Addressed lines 18-19: “Interviews were conducted with 13 ED patients and 37 caregivers, on the behalf of patients when unavailable.”

16-19: you addressed the age questions in the revisions, however I still find it slightly confusing. The mean ages are not clear. All patients had an average age of 33, but all interviewees were 38 years on average? This sentence seems out of place, as you switch back to the patients’ experiences afterwards. I suggest removing that sentence altogether*.*

*This was removed and helped to make space for other clarifications.*

Line 58-60. Much clearer description of the aims, using a more qualitative language than in the abstract
Thank you.

Line 61-108: appreciate the explanations and giving context to the reader. Well written and informative.
Thank you.

Methods:
- I suggest using sub-headings for the methods section to make it more reader friendly.

These have been added.

- Provide a brief statement at the beginning of the methods that outlines the study design and analysis approach. This will be especially beneficial for readers that are not well versed in qualitative research designs.

*This was added on lines 122-128: “To better understand the challenges rural Ugandans face in accessing and utilizing EC we purposefully sampled and interviewed patients presenting to the ED more than 12 hours after onset of chief complaint or their caregiver when the patient themselves was unavailable. Semistructured interviews addressed actions taken before seeking EC and delays once the need for EC was recognized. We purposefully interviewed a diverse sample of patient demographics and chief complaints. An interdisciplinary and multicultural research team conducted thematic analysis based on descriptive phenomenology, using the Framework method.”*

- You state in the title that a grounded theory approach was used, however that is a very specific iterative approach, that requires more details on how the approach was followed, vs an analysis based in phenomenology for example. Make sure you truly used a complete grounded theory approach, rather than utilizing certain elements based in grounded theory. Specify how the Framework Method helps to follow a grounded theory approach.

*This is a very valid insight. While a true grounded theory approach was the intention, we used more pragmatic approach which does not fully adhere to grounded theory. The mention of grounded theory has been removed throughout.*

Line 111: you justify the choice for the 12 hour time mark in your response letter, I suggest adding that here. It provides additional rigor to the methods.

*This was added in lines 125-127: “≥12 hours was set as a benchmark based on advice from the Ugandan clinicians working in the ED due to their observation that patients arriving outside of this timeframe are subjectively noted to have poor outcomes attributable, at least in part, to delayed presentation.”*

Line 114: information rich cases. Briefly state how you determined who’d have significant experience with challenges in EC access prior to having interviewed them. What were the characteristics the ED staff suggested you to include to meet this criteria?

*This was added on lines 139-140: “These conversations often focused on challenges with identifying the need for EC, obtaining EC from local facilities, finance, or transport.”*

Line 129-131: I am curious if severe illness of the patient was a common reason for caregivers to refuse the interviews. I imagine this could be a very stressful and potentially challenging interview for caregivers and patients to have. Where any resources put in place, in case they needed support?

*For exactly these reasons we did not approach caregivers when patients were severely ill. A description was added on lines 145-146: “Recruitment and interviews did not interfere with patients’ clinical care and were not conducted when the patient was severely ill but rather once they were stabilized, if at all.”*

Line 132-145: good description of the interview guide development. Was piloting of the questions performed? If not, what was the rationale?

*Added on lines 173 -175: “The guide was initially piloted with community members not seeking EC followed by approximately ten ED patients or their caregivers.”*

Line 146: briefly describe how the training in qualitative interview-based methods looked like. Didactics only, practice sessions, etc.

*Added on lines 177-179: “After didactic training in qualitative interview-based research methods by AP,* *the head research assistant (NC), a resident of Rukungiri District and native Runyankore/Rukiga and fluent English speaker, obtained practical training, experience and feedback while piloting the interview guide.”*

Lines 172- 183: the data saturation statement is still not clear. I understand the rationale about adding more interviews to have a broader demographic profile, but it still sounds like saturation was reached after 25 interviews and not after 50. How exactly was saturation determined? Provide more details on the type of saturation (thematic vs data saturation)

*Revised lines 204 -211 for clarity: After the first 25 interviews were conducted and analyzed, the data analysis team identified that no additional large-scale concepts of delay were emerging; thematic saturation was reached. The team convened to review subthemes across these first 25 interviews simultaneously and organize them, yielding the 4 themes described in Results. These themes and subthemes constituted a codebook, included as Appendix S2, facilitating coding of an additional 25 interviews completed to capture a broader sample of demographic groups and chief complaints across both dry and rainy seasons, given differences in EC accessibility and subsistence agriculture activities.*

Line 194: I appreciate switching the Tables 1 and 2. Much clearer

*Thank you for the useful suggestion.*

198: the distinction of “delay in ED presentation” and “Themes in Delay” is not very clear. Delay in ED presentation provides the quantitative explanation of delays, whereas themes in delay further explains and seeks to understands the delays.

*Although you understood and stated the difference perfectly this is a very helpful clarification to make. This was added on lines 237-240: “Delay in ED presentation provides the quantitative explanation of delays, whereas themes in delay — presented below — seek to further understand and explain these delays in ED presentation.*

220: A brief overview of themes and subthemes in table form or some visual could be helpful

*A very helpful idea. Table 4. Themes and subthemes in delay identified was added on lines 244 – 245:*

**Table 4. Themes and subthemes in delay identified**

| Theme | Subthemes |
| --- | --- |
| Cultural Factors and Limited Knowledge of Emergency Signs and Initial Actions to Take | Difficultly recognizing “danger signs”   - Patient’s condition becoming severe before identifying the need for and/or seeking EC - Perception of “cultural illness” |
| Use of Local Facilities for Stabilization and Advice Despite Perception of Inadequate Services | - Limited first aid or home care - Use of local facilities:  1. Assessable on foot or bicycle 2. For advice and symptom management 3. While gathering funds needed for EC 4. Despite perception of poor care at high cost  - Use of multiple facilities before seeking EC - Perception of delays in referral to EC |
| Lack of Resources to Cover the Direct, Indirect, and Opportunity Costs of EC | - Perception of EC as unaffordable - Need for funds for transport prior to seeking EC - Opportunity costs of EC |
| Inadequate Transportation Options, Especially at Night and in Inclement Weather | - Inadequate vehicles available for the patient’s condition - Dirt roads virtually unusable in heavy rain - Safety concerns when traveling at night |

239: Could you include the specific word used for “cultural illness” in the local dialects?

*I discussed this with Charles Ndyamwijuka and the Ugandan members of our research team all of whom are native speakers. There is no one specific word for “cultural illness” these were identified through patient or caregivers’ descriptions of the presumed etiologies and treatment strategies.*

259: Do you have information on what the maximum number of facilities visited before the ED presentation was?

*Unfortunately, I do not. We queried types of facility and estimated costs but did not ask for a specific number of facilities. The use of multiple facilities was a somewhat unexpected result and was identified as multiple interviews were compared.*

288: I understand you mentioned in your response letter that it is difficult to provide context on how much a dollar sum is in the Ugandan context, specifically in this region, where a national net income would likely be an overestimate. But I think it is important to emphasize somehow, that 150$ is a significant sum and potentially catastrophic healthcare expenditure. Maybe you could add some small section in the description of the setting (lines 68-69)

This is included on lines 80-81: “The vast majority of inhabitants are subsistence farmers (78%), and most lack regular access to money, especially between harvest seasons [6].”

324: change to past tense, check for consistency throughout

This was changed and the rest of the manuscript reviewed.

343: Discussion makes the objectives and planned next steps much clearer, positioning this paper in the ongoing research efforts. Good integration of the available literature.

Thanks!

Reviewer #2: (No Response)

Reviewer #3: I think you have addressed and covered the reasons for delay in careseeking of this area in Uganda well and got results that are in line with the Three Delays Model. These results are discussed thoroughly in the discussion as well as suggestions and thoughts on how to move forward are pointed out. With all studies there are limitations and I think you are aware of them and address them wisely.
